# 3D field-shaping lens using all-dielectric gradient refractive index materials

**DOI:** 10.1038/s41598-017-00681-z

**Published:** 2017-04-10

**Authors:** Tongyu Ding, Jianjia Yi, Haoyu Li, Hailin Zhang, Shah Nawaz Burokur

**Affiliations:** 1 State Key Laboratory of Integrated Services Networks, Xidian University, Xi’an, 710071 Shaanxi China; 2 School of Information Engineering, Jimei University, Xiamen, 361021 Fujian China; 3 Department of Biomedical Engineering, Stony Brook University, State University of New York, Stony Brook, New York 11794 USA; 4 LEME, EA 4416, Université Paris Nanterre, 92410 Ville d’Avray, France

## Abstract

A novel three-dimensional (3D) optical lens structure for electromagnetic field shaping based on spatial light transformation method is proposed at microwave frequencies. The lens is capable of transforming cylindrical wavefronts into planar ones, and generating a directive emission. Such manipulation is simulated and analysed by solving Laplace’s equation, and the deformation of the medium during the transformation is theoretically described in detail. The two-dimensional (2D) design method producing quasi-isotropic parameters is further extended to a potential 3D realization with all-dielectric gradient refractive index metamaterials. Numerical full-wave simulations are performed on both 2D and 3D models to verify the functionality and broadband characteristics of the calculated lens. Far-field radiation patterns and near-field distributions demonstrate a highly radiated directive beam when the lens is applied to a conical horn antenna.

## Introduction

Transformation Optics (TO) concept^1, 2^, known as a powerful and effective method to simultaneously control electromagnetic (EM) fields, has been widely used to exploit new classes of optical and electromagnetic devices. The best known design by TO approach is the invisibility cloak^3^. This work has pushed further forward the development of other conceptual and functional tools such as illusion devices^4–8^, waveguide bends and transitions^9–15^, lens antennas^16–23^, and so on. Nevertheless, both permittivity and permeability values tailored by traditional TO concept are inhomogeneous and anisotropic in general, leaving a large amount of devices unrealized experimentally. These designed structures sometimes present a real challenge for practical implementations and mostly require resonant artificial electromagnetic materials. To address this issue, metamaterial structures such as split ring resonators (SRR)^24^ and electric LC (ELC) resonators^25^, to name the most common, have been widely implemented in TO-based devices. However, the issue of limitations inherent to intrinsic absorption and narrowband properties still remains to be addressed.

To overcome the limitations of the resonant nature of metal-dielectric metamaterials, quasi-conformal transformation optics (QCTO) has been proposed to produce quasi-isotropic devices, including the carpet cloak^26^ and bent waveguides^27^. Hu *et al*.^28^ proposed an equivalence between coordinate transformation and spatial deformation by using Laplace’s equation with Dirichlet-Neumann sliding boundary conditions to determine the deformation of coordinate grids. This technique minimizes the anisotropy of the constitutive transformation medium, enabling potential realizations from non-resonant metamaterials. In particular, QCTO provides the possibility to work with arbitrary shapes and avoid singularities. Thus it has been applied to design arbitrary cloak^29^ and functional lenses^30–33^. Some optical devices were realized simply with dielectric materials and gradient refractive index metamaterials. Therefore, QCTO opens up new horizons for antenna designs in which the operation bandwidth is always a key consideration for potential industrial applications.

In a previous study, we have synthesized a transformed all-dielectric material and fabricated a 2D lens that is capable of restoring in-phase emissions from a conformal array of antennas so as to obtain performances similar to a linear one. In this work, we introduce such a design procedure to a 3D lens with a mushroom shape that can significantly enhance the directivity of a conical horn antenna. In horn antennas, such as feeds of parabolic satellite dishes, the radiating aperture of the horn is usually covered by dielectric radome transparent to radio waves for protection against weather conditions. The proposed cap lens, which can be potentially realized by all-dielectric gradient refractive index metamaterials, can be more compact than the classical radome and even allow obtaining an enhanced directivity. Here, Laplace’s equation is used to calculate the constitutive electromagnetic parameters of the transformation medium. It is shown that the principle of the transformation media corresponds in fact to specific boundary conditions. Both two- and three-dimensional full wave simulations based on finite element method (FEM) are performed over a wide frequency range spanning from 7 to 13 GHz to validate the broadband performances and functionality of the proposed method. Far-field antenna patterns and near-field distributions are presented to demonstrate the directivity enhancement properties of the calculated lens.

## Results

### Theoretical modeling of the lens

The scalar two-dimensional Helmholtz equation is form invariant with respect to coordinate transformations, and can be regarded as equivalent to a conformal mapping. Thus QCTO is an approximate solution of minimizing the anisotropy for general boundary conditions.

**Figure 1:**
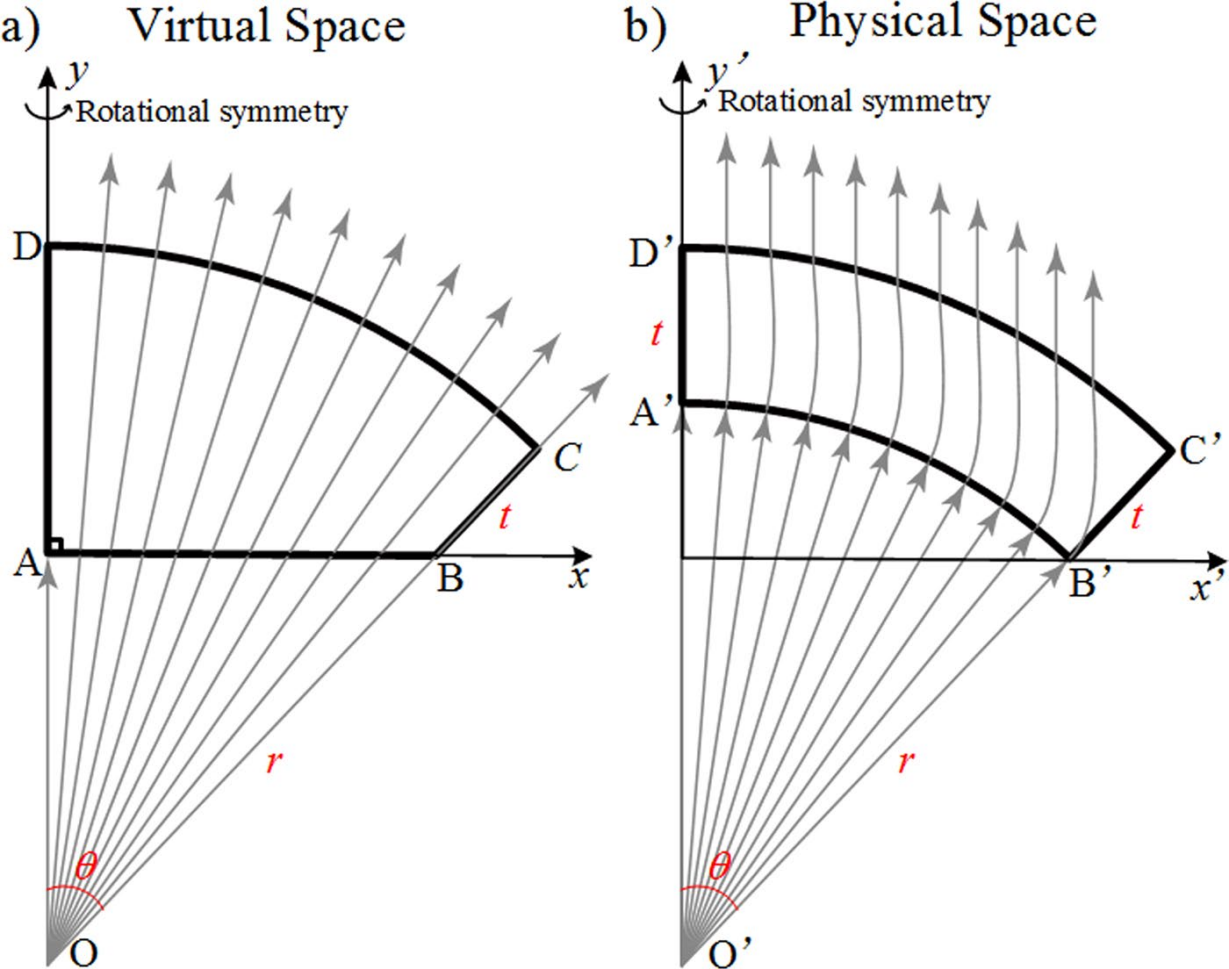
Illustration showing the space mapping from the virtual space to the physical space for the field shaping lens where the gray arrows represent the ray tracing.

The virtual and physical spaces are respectively denoted by (*x*, *y*) and (*x*′, *y*′). The air filled virtual space ABCD is presented as illustrated by the schematic principle in Fig. 1(a). *CD* is an arc of circle with center *O* and radius is *r* + *t*, where the central angle in radians is written as *θ*. Segments of *BC* and *DA* are along the direction of radius. The length of *BC* is taken to be *t*, and *AB* is perpendicular to *AD*. The real physical space *A*′*B*′*C*′*D*′ filled by transformed medium is illustrated in Fig. 1(b). Similarly, *A*′*B*′*C*′*D*′ is an annular sector whose central angle in radians is also *θ* and its center is taken as *O*′. *O* and *O*′, *B* and *B*′, *C* and *C*′, *D* and *D*′ share the same location respectively. We consider sector *O*′*B*′*A*′ as half of the 2D conical horn antenna. The cylindrical emission on the edge of arc *B*′*A*′ is transformed to the horizontal one, as arc *A*′*B*′ is transformed to segment *BA*.

The designed model is based on quasi-conformal transformation optics (QCTO) and achieved by solving Laplace’s equation. For fields’ equivalence at the outer boundaries with the virtual space, Neumann and Dirichlet sliding boundary conditions are set at the edges of the cap lens:

1 $$\begin{array}{c}x|{}_{{B}^{^{\prime} }{C}^{^{\prime} },{C}^{^{\prime} }{D}^{^{\prime} },{D}^{^{\prime} }{A}^{^{\prime} }}=x^{\prime} ,\quad \hat{n}\cdot \nabla x|{}_{{A}^{^{\prime} }{B}^{^{\prime} }}=0\\ y|{}_{{A}^{^{\prime} }{B}^{^{\prime} }}=0\,,\quad y|{}_{{B}^{^{\prime} }{C}^{^{\prime} },{C}^{^{\prime} }{D}^{^{\prime} }}=y^{\prime} ,\quad \hat{n}\cdot \nabla y|{}_{{D}^{^{\prime} }{A}^{^{\prime} }}=0\end{array}$$

Previously, Li and Pendry suggested that the small anisotropy can be ignored if the deformation is small enough^25^. Considering the polarization of the excitation, the properties of the intermediate medium can be further simplified as:

2 $$\varepsilon =\frac{{\varepsilon }_{r}}{{\rm{\det }}({J}^{-1})},\quad \mu =1$$

where $$J=\frac{\partial {x}^{i}}{\partial {x}^{{i}^{^{\prime} }}}$$

It is clear that the properties of the medium are isotropic in the lateral (*x*-*y*) plane. It is worth noting that the 2D design can easily be extended into a 3D model due to its rotational symmetric feature. Quasi-isotropic medium properties allow non-resonant metamaterials implementation, which renders a broadband device physically realizable. All-dielectric gradient refractive index metamaterials are applied in the latter design procedures.

**Figure 2:**
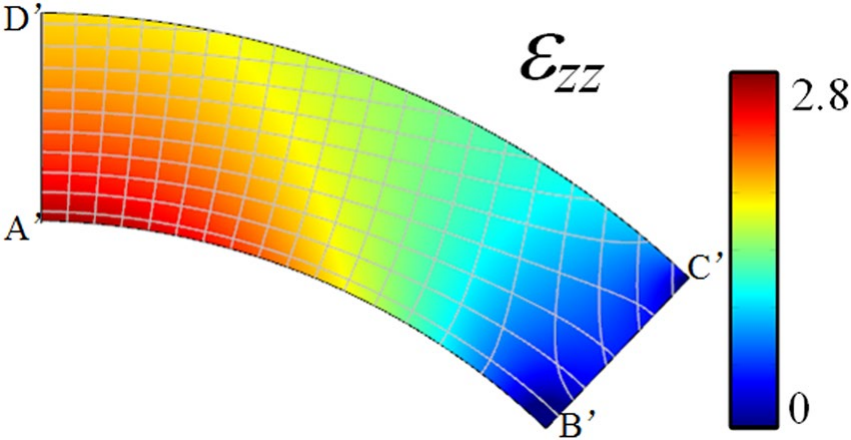
The calculated permittivity (*ε*_zz_) distribution varies from 0 to 2.8.

The distribution of permittivity (*ε*_zz_) perpendicular to the *x-y* plane in the physical domain is presented in Fig. 2. The range of *ε*_zz_ is related to the deformation between the physical space and the virtual space. We consider the initial parameters as *t* = 4 cm, *r* = 14.5 cm and *θ* = 0.761 rad. In this case, the permittivity distribution ranges from 0 to 2.8. To refrain from using resonant metamaterials and to support a potential all-dielectric realization process, we have to consider *ε*_zz_ values below 1 as unity.

### Numerical validation

In this section, in order to design and characterize the proposed directivity enhancement lens, a sequence of numerical simulations based the finite element method is performed. 2D models are verified by using commercial software Comsol Multiphysics^34^ with typical initial conditions and values of the model. Scattering boundary conditions are set around the computational domain. The conical horn antenna is modeled by a sector of radius *r* = 14.5 cm with its central angle chosen to be 2*θ* and perfect electric conductor boundary conditions are set at the two arms of the sector. An out-of-plane line source is located close to the center of the sector, and represented by a dot. The electric field of the exciting sources is polarized along the *z*-direction. The annular sector shaped lens area is assigned by the isotropic medium properties obtained from the solution of the Laplace’s equation illustrated in Fig. 2. The lens is considered to be 4 cm thick.

**Figure 3:**
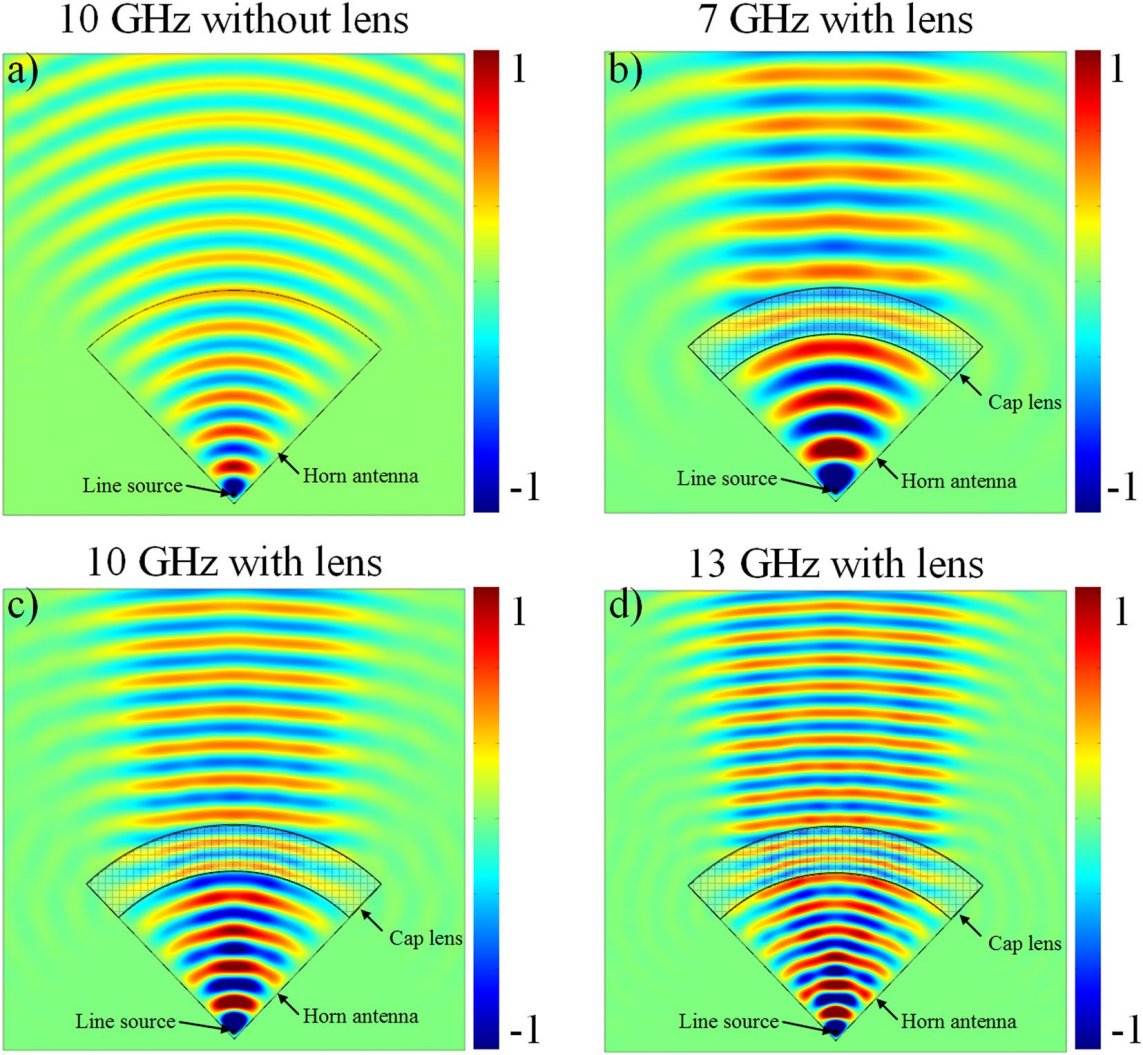
Calculated 2D electric field distribution of (**a**) conical horn antenna at 10 GHz, and conical horn antenna with cap lens at (**b**) 7 GHz, (**c**) 10 GHz and (**d**) 13 GHz.

In order to examine the reliability of the proposed model, simulations are performed to predict the evolutions of the electric field distribution, as shown in Fig. 3(b,c and d) at 7 GHz, 10 GHz and 13 GHz respectively. An emission from a regular conical horn antenna without propagating through the lens is presented in Fig. 3(a) as a reference. It can be noticed that in the case of the conical horn antenna alone, the wavefronts are quasi-cylindrical. As it can be clearly observed in Fig. 3(b,c and d), the outgoing waves of the horn antenna in presence of the lens present planar wavefronts and therefore a directive emission, at the three tested frequencies.

**Figure 4:**
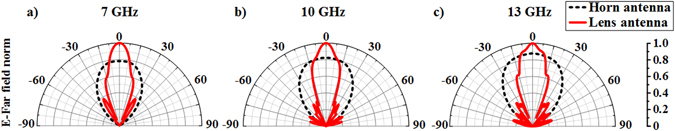
Normalized antenna radiation patterns showing the directionality enhancing performances of the cap lens antenna system at (**a**) 7 GHz, (**b**) 10 GHz and (**c**) 13 GHz.

We also examine the far field radiation patterns of both the horn antenna alone and the horn lens antenna system, and the corresponding results are illustrated in Fig. 4(a,b and c) at the tested frequencies. The black dashed curves represent the radiation patterns of the horn antenna alone, while the red solid curves represent the radiation patterns of the horn lens antenna structure. As it can be observed, the lens enhances the directivity of the horn antenna by narrowing the primary lobe and increasing its intensity.

### Potential 3D discrete lens validation

**Figure 5:**
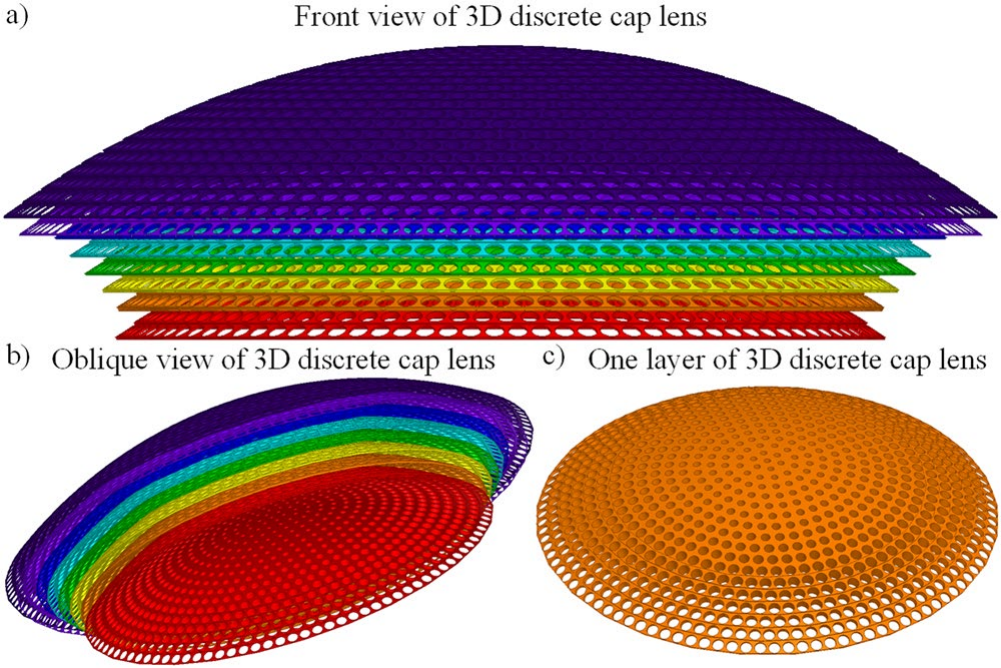
Design of the 3D discrete lens composed of 8 layers.

A 3D discrete lens is designed for further realistic full-wave numerical simulations. The permittivity profile of such lens is shown in Fig. 2. According to effective medium theory, if the operating wavelength is large enough with respect to the size of the unit cell, a composite material can be considered to be isotropic and homogenous. We therefore propose a discrete lens model, as presented in Fig. 5(a). The profile is divided into eight different layers, each represented in a different color. Each layer is composed of several circular arrangements of cubic cells, as shown in Fig. 5(c), and as a result, the whole lens is composed of 158 different circular rings containing a total of 9366 cubic cells. The cubic cells within each circle share the same structure. The discrete permittivity of each cell is constant and is equal to the average permittivity within the cell.

To make the lens operate over a broad frequency range, the lens is realized from non-resonant all-dielectric metamaterial cells. Air holes in a dielectric host medium of relative permittivity *ε*_*r*_ = 2.8 is therefore considered. Such type of air-hole structures have been successfully implemented in gradient index materials for applications to lenses^35^. Other metal-dielectric metamaterial structures have also been used as gradient index materials for high-directivity lenses in horn antennas^36^.

**Figure 6:**
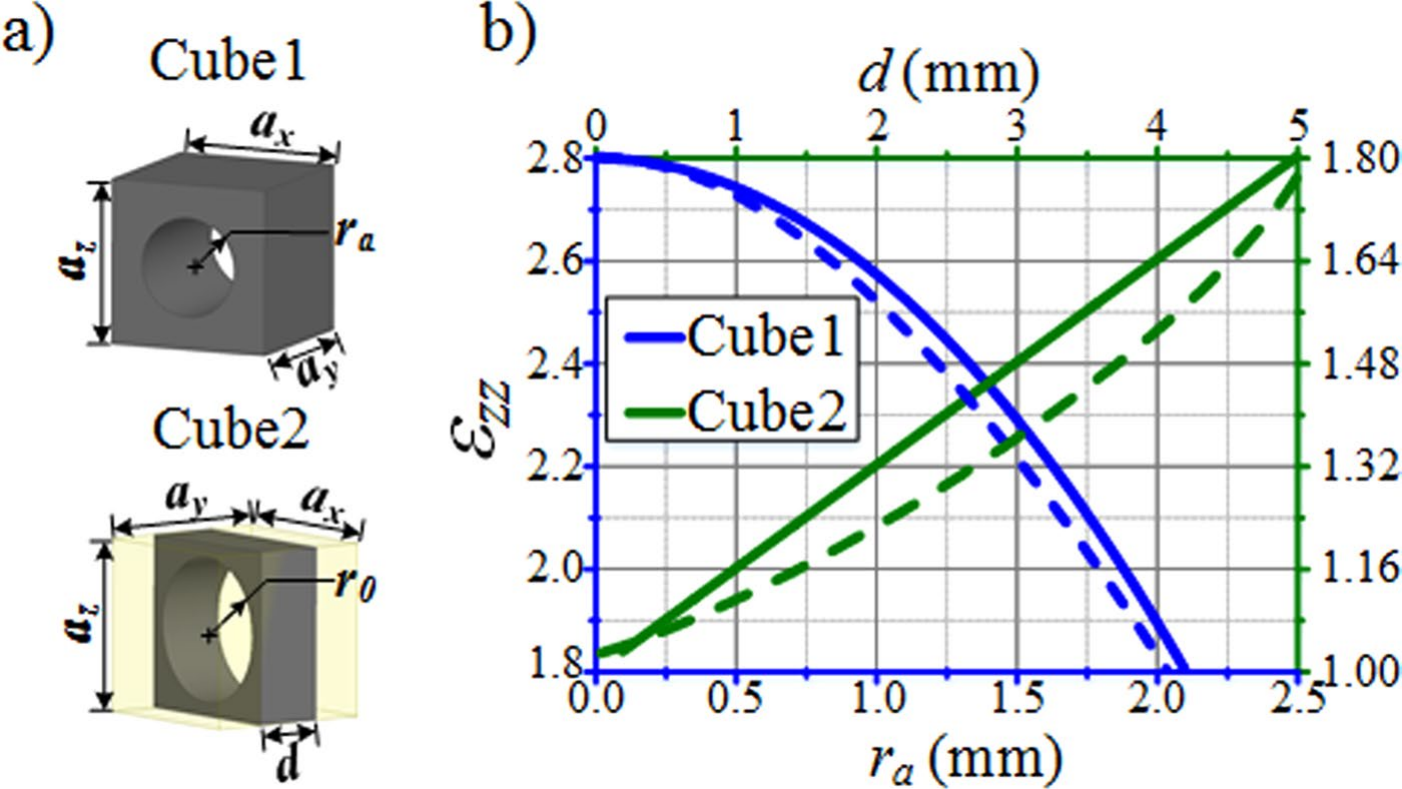
(**a**) Unit cells that can be used to implement the lens. (**b**) Effective permittivity of the cells composed of an air hole in a dielectric host medium. A parametric analysis is performed to extract the effective permittivity value according to the radius *r*_*a*_ of the air hole for cube 1(with *d* equal to *a*_*y*_) and to the thickness *d* for cube 2. The continuous traces correspond to approximate values calculated using the mixing formula and the dashed traces correspond to accurate values retrieved from homogenization procedure.

Suppose that two materials are mixed together, the effective parameter can be approximated by:

3 $${\varepsilon }_{e}={\varepsilon }_{a}{f}_{a}+{\varepsilon }_{h}{f}_{h}$$

where *ε*_a_ = 1 and *f*_a_ and *f*_h_ are the volume fraction of the air holes and the host material, respectively. By adjusting the volume fraction of the air holes in the dielectric host medium, the effective permittivity of the cell can be engineered at will. The design of the 3D cap lens is composed of two types of unit cells, as shown in Fig. 6(a). A parametric analysis is performed to extract the effective permittivity value according to the radius *r*_a_ of the air hole for cube 1 (with *d* equal to *a*_y_) and to the thickness *d* for cube 2. The solid traces correspond to approximate values calculated using the mixing formula and the dashed traces correspond to accurate values retrieved from homogenization procedure by using the inversion method^37^. Figure 6(b) shows the typical values of the engineered effective permittivity. Thus, cubic cells in the different regions of the discrete lens can be readily prepared for implementation.

**Figure 7:**
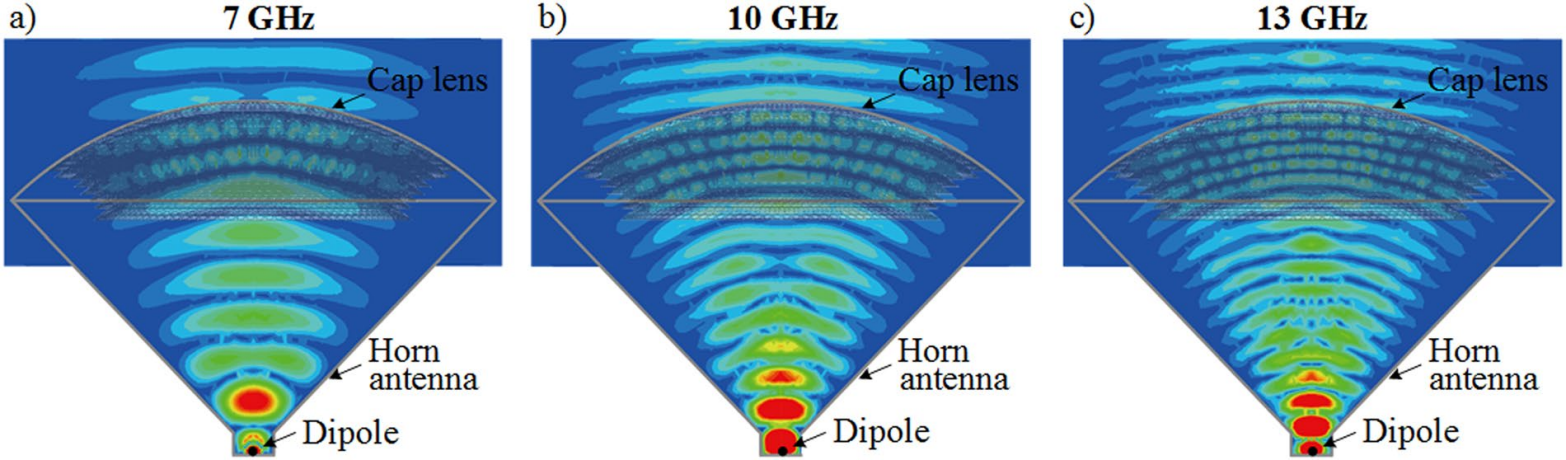
Simulated electric near field distributions of 3D discrete lens in the *x-y* plane at (**a**) 7 GHz, (**b**) 10 GHz and (**c**) 13 GHz.

Full-wave simulations using Ansys HFSS^38^ are performed to numerically simulate and validate the functionality of the 3D cap lens. A dipole element is applied as a primary feeding source of the conical horn antenna. The proposed cap lens is placed in the aperture of the horn. Calculated near-field distributions are presented in Fig. 7. Based on the results in Fig. 7, it can be concluded that a directivity enhancement is produced by the lens. It is also worth noting that the resulting cylindrical wavefronts emitted from the horn antenna become planar after transmitting through the lens.

**Figure 8:**
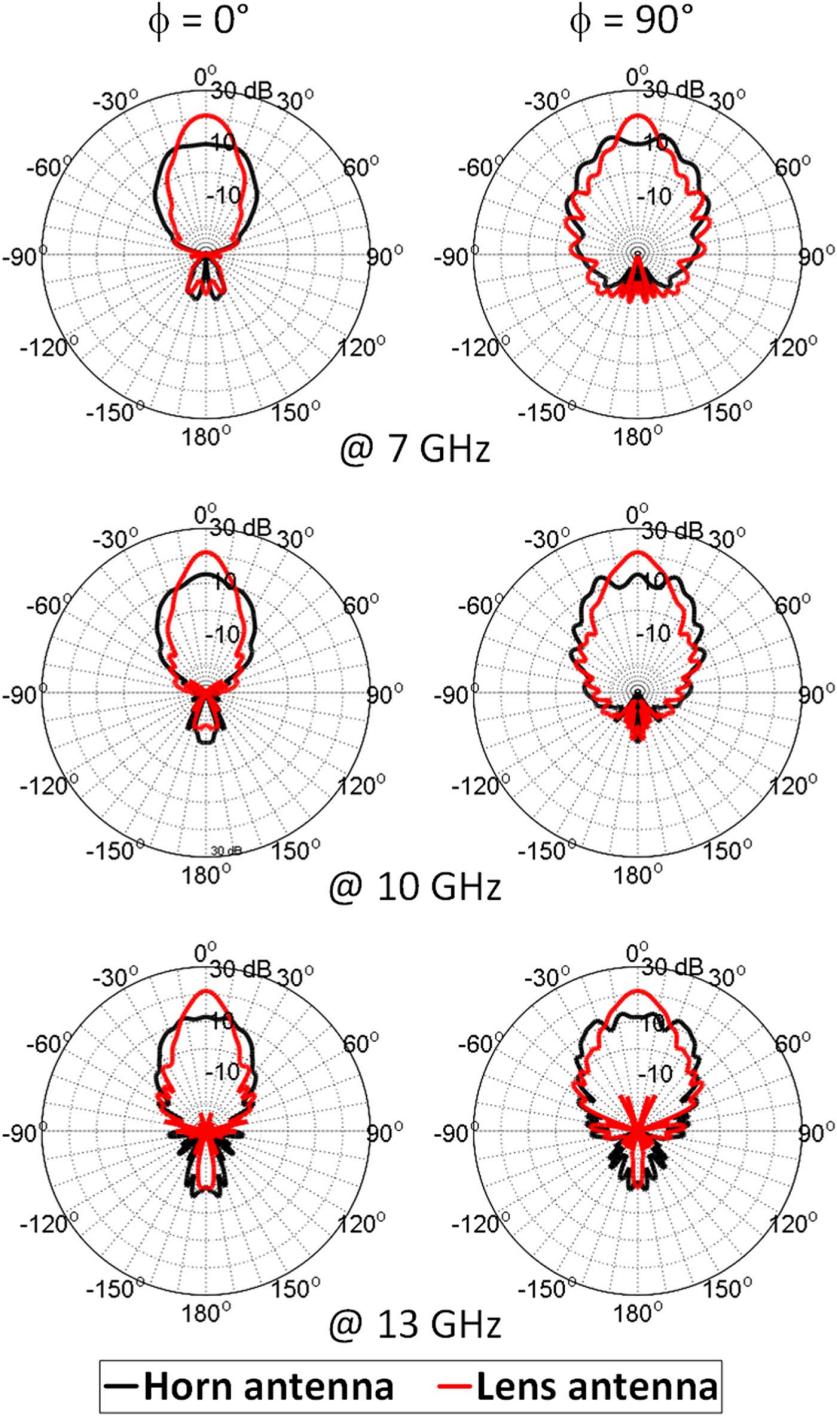
Simulated electric far field distribution of the conical horn antenna alone and the horn antenna with the 3D discrete lens in the *x-y* plane at 7 GHz, 10 GHz and 13 GHz.

**Figure 9:**
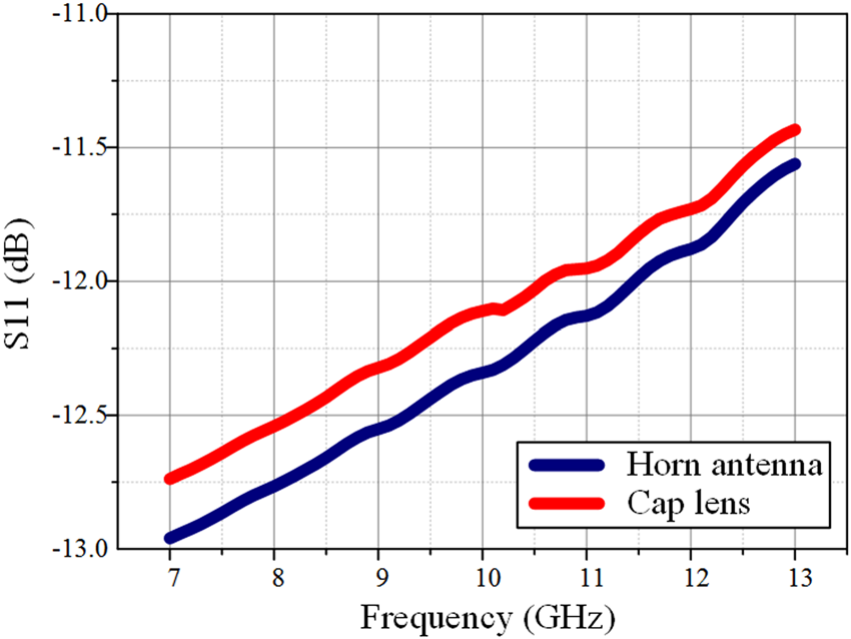
Simulated *S*_11_ coefficient distribution of the conical horn antenna alone and the horn antenna with the 3D discrete lens.

Far-field reports of the directivity are also presented in Fig. 8 over a broad frequency range. In presence of the dielectric lens, the primary lobe radiated from the horn antenna is narrowed and a higher directivity is observed, confirming the fact that the lens is able to produce a directive emission with less dispersion. Figure 9 presents the *S*_11_ coefficient of the lens antenna system. The cap lens introduces negligible losses to the horn antenna, which guarantees the impedance matching of the whole system.

## Discussion

In summary, a novel concept design procedure of a compact all-dielectric conformal lens has been presented and numerically validated. The lens is able to enhance the directivity of a conical horn antenna on a broad frequency range. The lens has been numerically characterized in the microwave region from 7 GHz to 13 GHz. The near-field distributions have shown that such a lens is able to flatten the cylindrical wavefronts from a horn antenna into planar ones in order to produce a highly directive radiation pattern. Furthermore, far-field antenna patterns have shown directive emissions from the horn antenna over the tested frequency range. The method proposed in this paper is low-cost and can be easily implemented at microwave frequencies. Such all-dielectric devices present real potential applications in airborne and communication systems. The method is general and can be freely applied to any 2D and 3D shapes.
